# Supplementary material for: Tunable quantum anomalous Hall effects in ferromagnetic van der Waals heterostructures
Source: Natl Sci Rev. 2023 May 25;11(3):nwad151. doi: 10.1093/nsr/nwad151 (PMC10833467; doi:10.1093/nsr/nwad151)
Supplement: nwad151_Supplemental_File [file nwad151_supplemental_file.docx]

**Supplementary Materials of “Tunable Quantum Anomalous Hall Effects in Ferromagnetic van der Waals Heterostructures”**

Feng Xue^1,2^, Yusheng Hou^3^, Zhe Wang^4^, Zhiming Xu^2^, Ke He^1,2,5^, Ruqian Wu^6^, Yong Xu^2,5,7,8, *^, Wenhui Duan^1,2,5,9, *^

**Part I. Band structures of Bi bilayer (BL) and MnBi_2_Te_4_ septuple (SL) layer.**

Bi BL is a nonmagnetic insulator with a band gap of 0.45 eV (0.46 eV) by GGA+U (HSE06) calculation [Fig. S1(a) (Fig. S1(c))]. MnBi_2_Te_4_ SL is a ferromagnetic (FM) semiconductor with a band gap of 0.19 eV (0.56 eV) by GGA+U (HSE06) calculation [Fig. S1(b) (Fig. S1(d))].


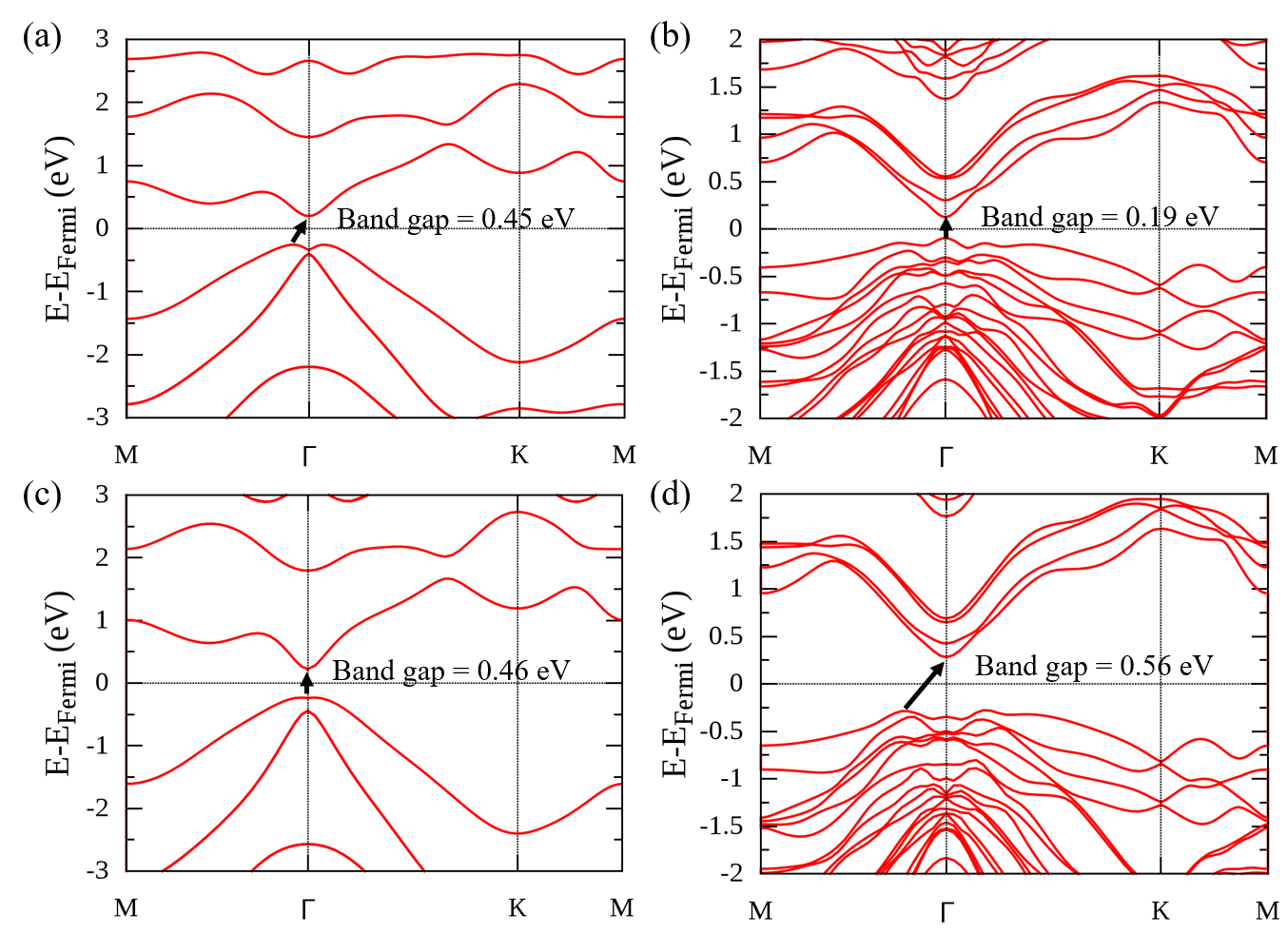


Figure S1. The calculated GGA+U+SOC band structures of (a) Bi BL and (b) MnBi_2_Te_4_ SL, respectively. (c) and (d) are corresponding HSE06 + SOC band structures. Values of band gap are shown.

The optimized lattice constants of MnBi_2_Te_4_ SL and Bi BL with spin-orbit coupling (SOC) are 4.37 Å and 4.38 Å, respectively. The magnetic exchange parameters are obtained by mapping the DFT total energy to the spin Hamiltonian $H=-J\sum_{<ij>} S_{i}\cdot S_{j}$, where $<ij>$ indicates the first nearest neighbors. Here, a $2\times1$ supercell is adopted to extract the magnetic exchange parameter *J*, with positive and negative values representing FM and antiferromagnetic (AFM) interactions, respectively. For FM and AFM configurations, the energies are given as $E_{FM}=-6J$ and $E_{AFM}=2J$, respectively. The magnetic anisotropy energy (MAE) is expressed as $MAE=E_{x}-E_{z}$, with positive and negative values representing in-plane and out-of-pane magnetization, respectively.

Table S1. The structural and magnetic parameters of Bi BL, MnBi_2_Te_4_ SL and Bi/MnBi_2_Te_4_ heterostructure. The lattice constant (*a*), exchange interaction parameter (*J*), magnetic anisotropy energy (MAE) and total magnetic moment ($M_{s}$) are listed.

| **Table S1 Structural and Magnetic parameters** | | | | |
| --- | --- | --- | --- | --- |
| System | *a* (Å) | *J* (meV) | MAE (meV) | $M_{s}$ (μB) |
| Bi | 4.38 | - | - | - |
| MnBi_2_Te_4_ | 4.37 | 0.97 | 0.25 | 5.0 |
| Bi/MnBi_2_Te_4_ | 4.38 | 1.08 | -0.15 | 5.0 |

**Part II. Six high-symmetry alignments and relative energies of Bi/MnBi_2_Te_4_ heterostructures.**

Figure S2 shows six stacking configurations of the Bi/MnBi_2_Te_4_ vdW heterostructure. The top layer is a single Bi (111) BL, with two sublattices of different height Bi atoms forming a buckled honeycomb structure. The bottom layer is MnBi_2_Te_4_ SL, consisting of Te-Bi-Te-Mn-Te-Bi-Te SL. The relative energy of different alignments is displayed in Table S2, from which we can know that the P1 configuration is the energetically most stable.


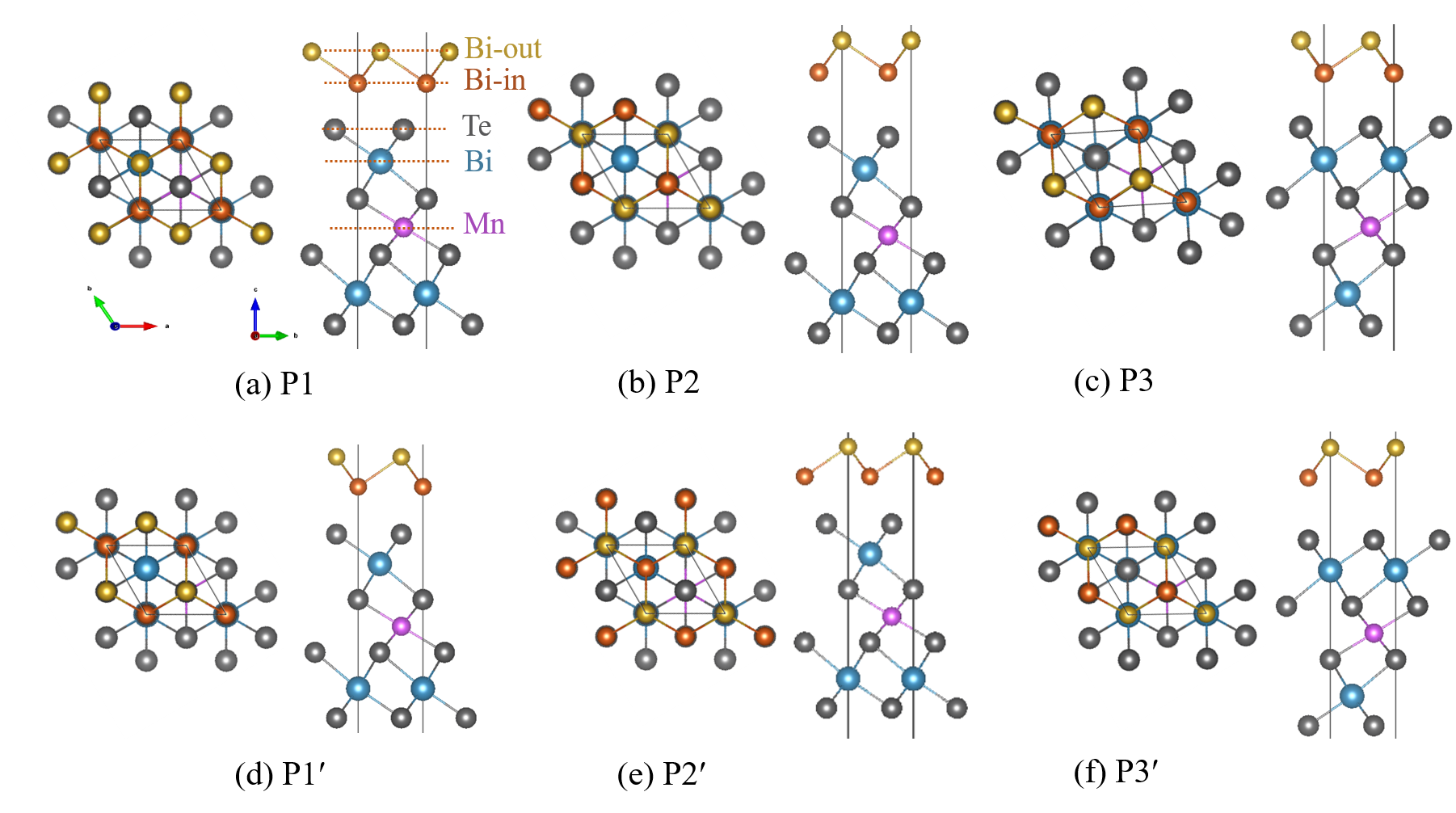


Figure S2. Six different stacking configurations of Bi/MnBi_2_Te_4_ vdW heterostructures. Depending on the position of the inner Bi atoms of Bi ML relative to interface atoms of MnBi_2_Te_4_, several configurations can be distinguished as follows: (a) Bi-in atoms sit on the hollow site of interface Te atoms; (b) Bi-in atoms are directly above the interface Te atoms; (c) Bi-in atoms are directly above the interface Bi atoms. (d)-(f) are similar to (a)-(c) but obtained by twisting the Bi BL by 60°.

Table S2. The relative energies of different alignments, where the energy of configuration P1 was set as the energy reference.

| **Table S2 Relative energies of six stacking configurations** | | | | | | |
| --- | --- | --- | --- | --- | --- | --- |
| Stacking | P1 | P2 | P3 | P1′ | P2′ | P3′ |
| Energy (meV) | 0 | 187.4 | 55.9 | 15.1 | 31.0 | 194.6 |
| Energy with SOC (meV) | 0 | 209.9 | 65.7 | 39.8 | 44.5 | 225.6 |

**Part III. MAE and HSE06 band structure of P1 configuration of Bi/MnBi_2_Te_4_ heterostructure.**

**
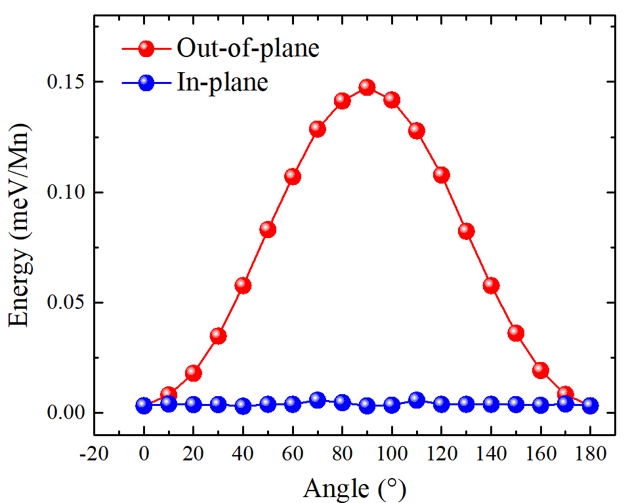
**

Figure S3. The MAE of Bi/MnBi_2_Te_4_ heterostructure, where the red and blue lines mean that the spin rotates in *x*-*z* and *x*-*y* planes, respectively.


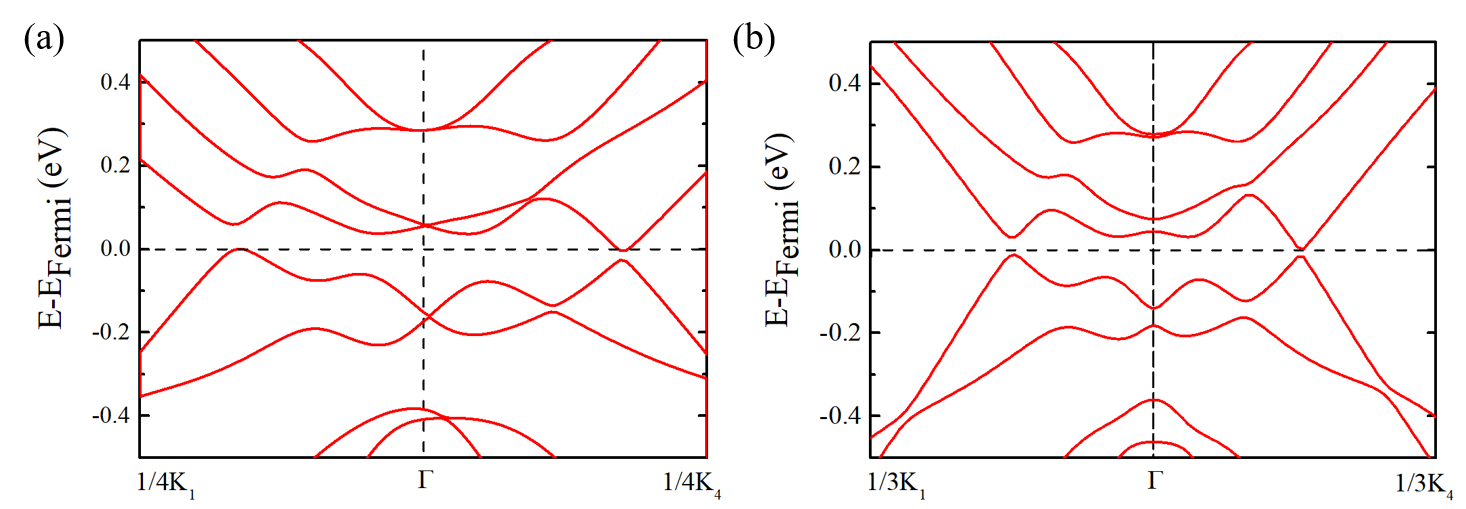


Figure S4. HSE06 + SOC band structures for (a) in-plane magnetization and (b) out-of-plane magnetization.

**Part IV. Angle dependence of QAHE for in-plane magnetization**


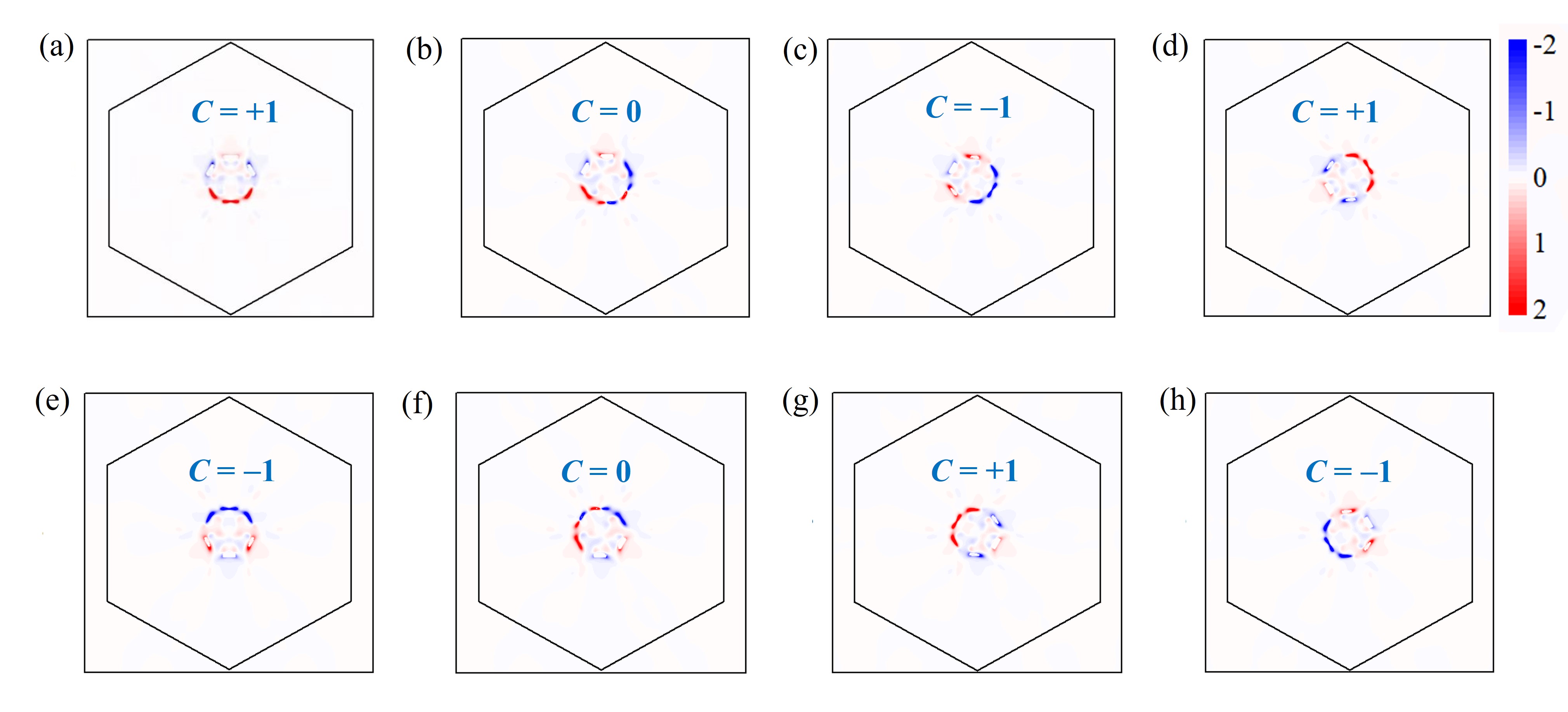


Figure S5. (a)-(h) The distribution of Berry curvature in the first BZ and the Chern number of P1 configuration with in-plane magnetization along *ϕ* = 0°, 30°, 60°, 120°, 180°, 210°, 240°, 300°. The color bars are shown in unit of 10^3^ Bohr^2^.


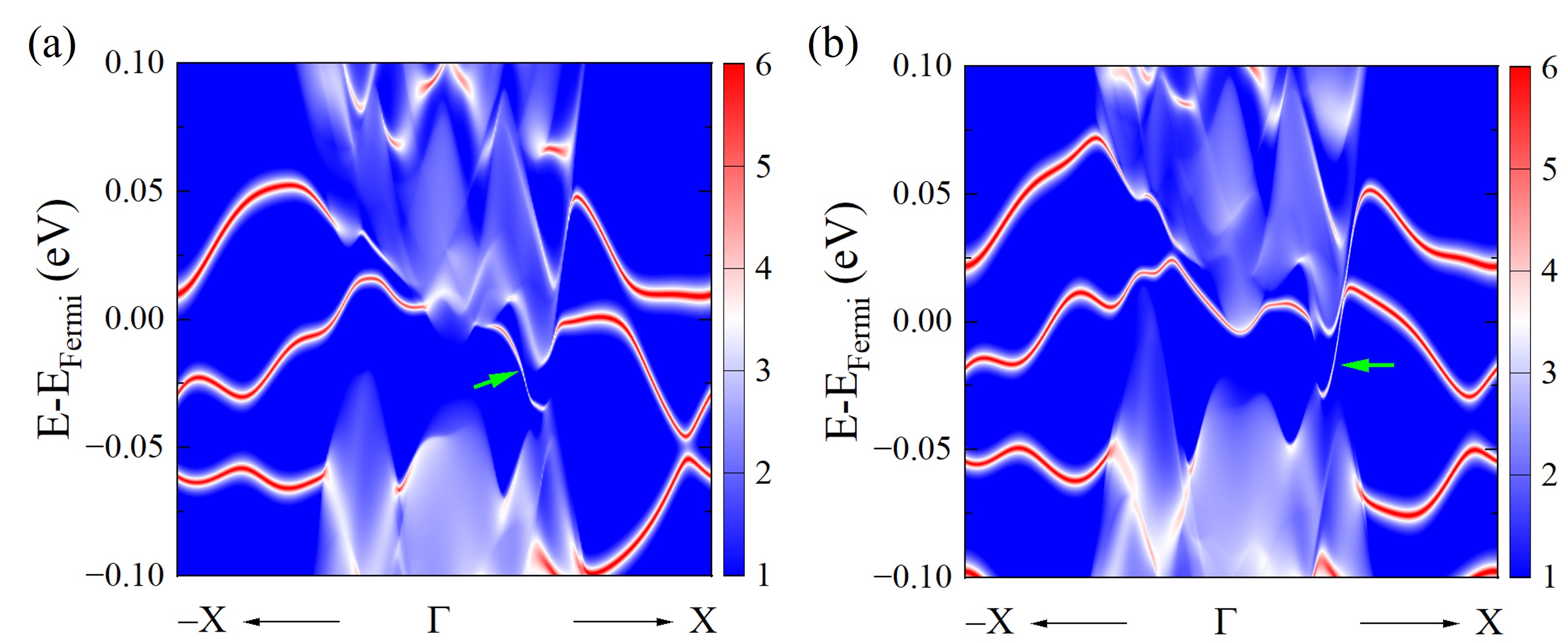


Figure S6. The edge states (highlighted by green arrows) of P1 configuration for in-plane magnetization along (a) 0° and (b) 60°. The $\left( 1\bar{1}0 \right)$ direction of Bi/MnBi_2_Te_4_ is studied.

**Part V. Strain effects on OPM-QAHE**


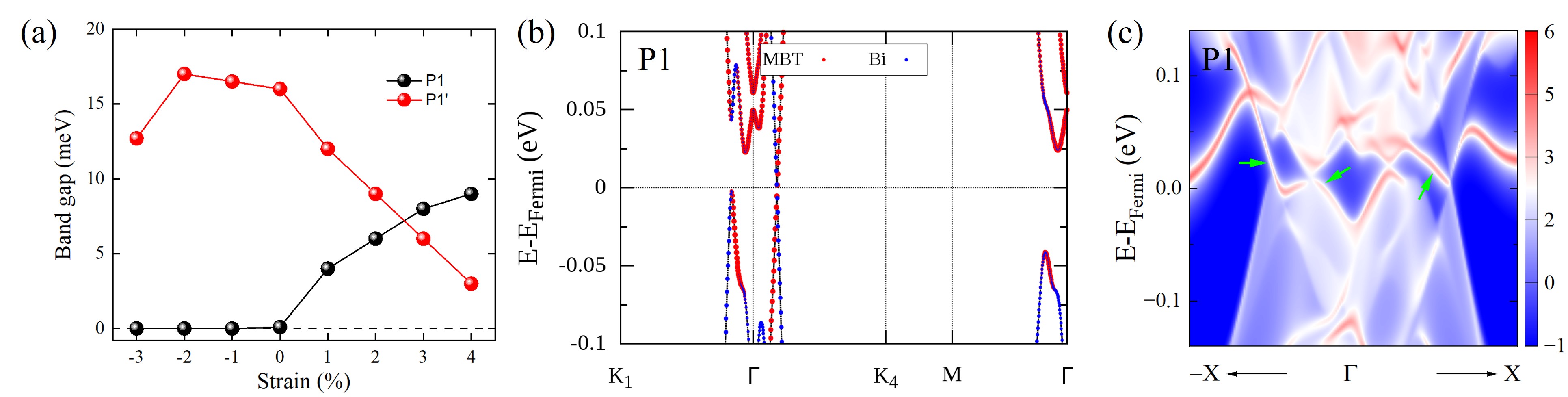


Figure S7. Topological properties of P1 configuration of Bi/MnBi_2_Te_4_ with an out-of-plane magnetization when no strain is applied. (a) The global band gap of the P1 and P1′ configurations as a function of strain fixing the magnetization along the out-of-plane direction. The positive and negative strains denote tensile and compressive strains, respectively. (b) DFT calculated band structure for the P1 configuration. (c) The chiral edge states (highlighted by green arrows) of Bi/MnBi_2_Te_4_ with the P1 configuration, where the (110) direction is studied.
